# Physiotherapeutic Rehabilitation of a Patient With a Comminuted Displaced Iliac Fracture and Superior and Inferior Pubic Rami Fractures: A Case Report

**DOI:** 10.7759/cureus.28709

**Published:** 2022-09-02

**Authors:** Pratik R Jaiswal, Madhu G Lakhwani, Pratik A Phansopkar

**Affiliations:** 1 Department of Musculoskeletal Physiotherapy, Ravi Nair Physiotherapy College, Datta Meghe Institute of Medical Sciences, Wardha, IND

**Keywords:** rehabilitation protocol, physiotherapy, pubic rami, iliac blade, acetabular rim fracture

## Abstract

Ilium fractures, which commonly advance from the iliac crest to the greater sciatic notch, are high-energy pelvic fractures that are frequently unstable. The general course of management for this injury is conservative, although cases of substantially displaced have been described that warranted surgical intervention. Many conditions, including decreased mobility, structural alterations in the joints, and discomfort, might make people more vulnerable to falls while waiting for hip or knee surgery. This can have an effect on both preoperative and postoperative functioning. The goal of surgical treatment for these individuals is to return them back to their prefracture state. This article presents the case of a 30-year-old male who was obtained a dash injury while riding a motorbike. He was diagnosed by an orthopedic surgeon with right-sided iliac blade fracture extending towards sacroiliac joint with right-sided sacral ala fracture and superior pubic rami fracture extending toward iliopectineal line and right-sided inferior pubic rami fracture (Tile Classification Type B3). He was operated via open reduction and internal fixation (ORIF) with osteosynthesis plating was done. Following surgery, the patient was dependent and his daily living was hampered. However, physiotherapy intervention improved the patient's pain and physical functioning and he gained independence in carrying out daily activities.

## Introduction

Slip-induced injuries account for 87% of hip fractures, which are expensive to treat and cause great suffering. These fractures commonly result in admittance to hospitals that provide specialized care [1]. Fractures resulting from accidents can cause in diminished freedom and functioning, limited mobility, lost confidence in one's ability to move, and lower quality of life [2]. Age-related bone resorption and trauma, most often from minor falls, are the main factors that lead to the majority of these fractures. Road accidents and falls from great heights are additional causes [3]. For the efficient eradication of infection, surgical excision of injured tissues and extraction of foreign particles is essential. Creating a stabilized fracture site, controlling the dead space, and systematic antibiotic therapy are equally important [4]. It is essential to properly realign and rebuild the shattered pieces. The best course of action for managing proximal tibia injuries is operative stabilization [5]. Intramedullary nailing, minimally invasive plate osteosynthesis (MIPO), open reduction and internal fixation (ORIF), and external fixation are among the prevalent surgical techniques [6]. The treatment's prognosis may be adversely affected by displacement, bone loss, soft tissue damage, infection, and related to numerous injuries [7]. The benefits of operational management include early mobilization and a shorter hospital stay. Additionally, it results in a decrease in systemic and local problems such as malunion and nonunion [8]. Following surgery, restricted weight bearing has indeed been linked to a longer healing time and a higher chance of developing postoperative problems. With the help of physiotherapy, early mobilization without limits and complete load bearing seems to enhance the adaptive postoperative result [9].

## Case presentation

Patient information

This case report details the treatment and follow-up of a 30-year-old man who was rushed to the emergency department after he met with a road traffic accident while riding a bike. He sustained injuries on his face and right hip and was unable to bear weight on his right leg. The patient was managed with suturing over lacerated wounds at a primary care unit. Then he was immediately rushed to a hospital where X-rays and MRI were performed (axial sections of the hip joints were taken without administration of intravenous contrast), and on that basis, an orthopedic surgeon diagnosed the right-sided hip fracture. There he underwent an operative procedure in which open reduction and internal fixation with plate osteosynthesis were performed. Following this, the patient started complaining of aches and decreased mobility of his right lower limb. The patient had no history of chronic diseases or psychological impairments.

Findings and Impression of Investigations

X-ray and MRI examination revealed that there were displaced fractures of the right pelvic bone involving the ilium and pubic bone. Superiorly fracture line extended to the left iliac blade and medially it involved the iliac part of the right sacroiliac joint. Linear displaced fracture of the superior and inferior pubic rami on right side was seen. A comminuted, minimally displaced fracture of the right sacral body and sacral ala was present, with intra-articular extension into the right sacroiliac joint.

Diagnosis

The patent suffered from right-sided iliac blade fracture extending towards sacroiliac joint with right-sided sacral ala fracture and superior pubic rami fracture extending toward iliopectineal line and right-sided inferior pubic rami fracture (Tile Classification Type B3).

**Figure 1:**
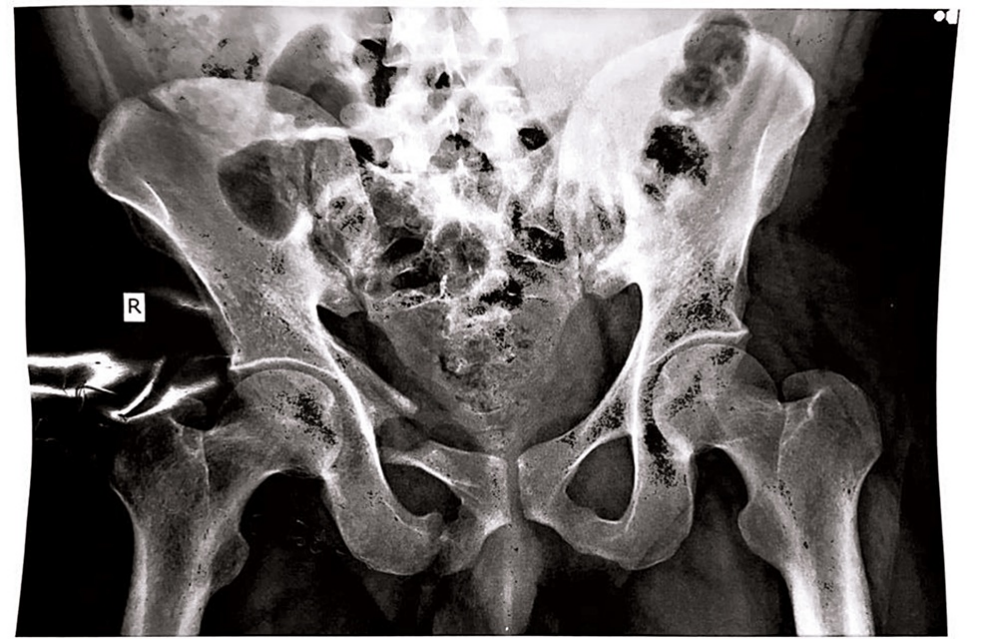
X-ray of the patient's hip

**Figure 2:**
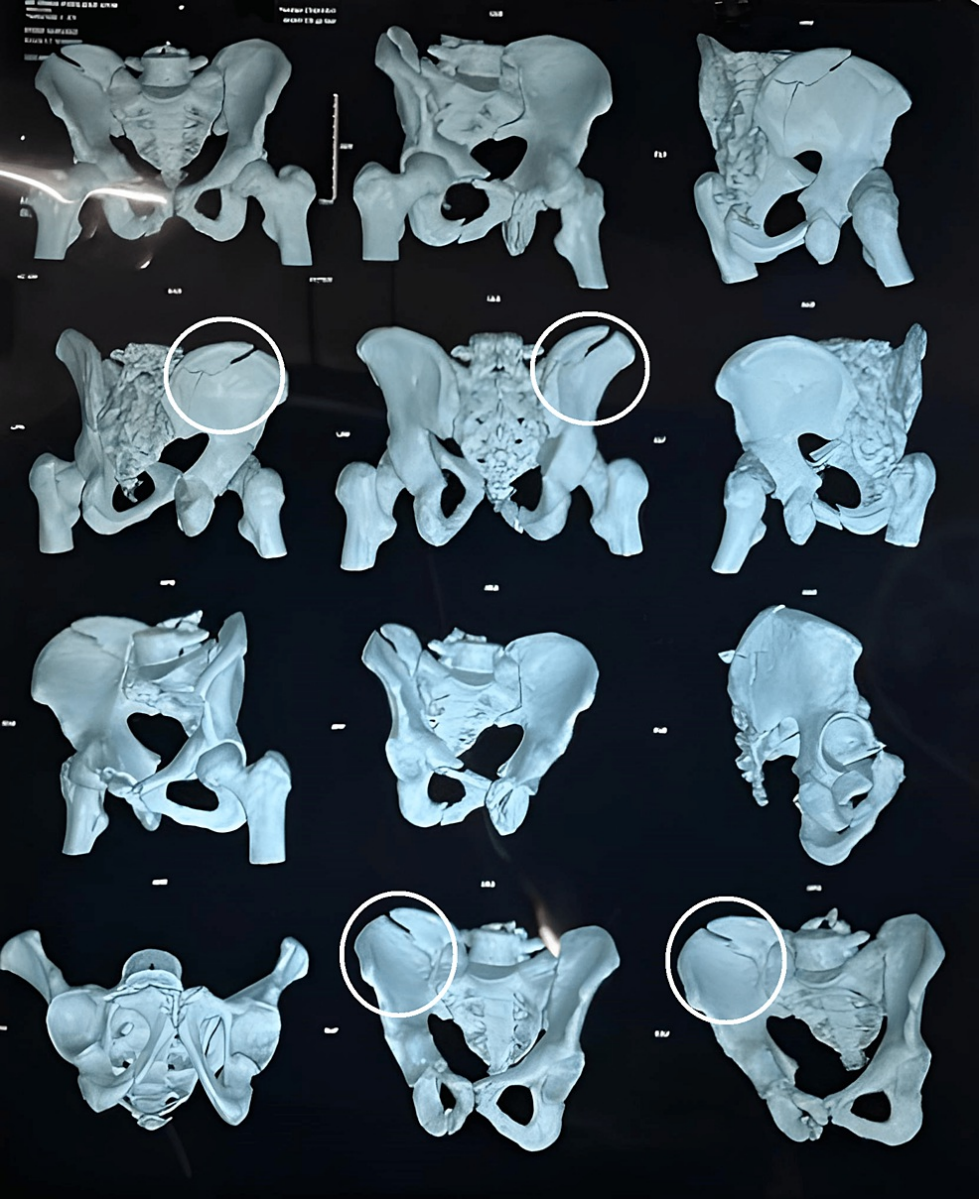
MRI of the patient's hip, with fractures part circled

Clinical findings

The patient was alert and well oriented in place, person, and time during the general examination. The patient's hemodynamics were normal: he was afebrile, BP was 130/78 mmHg, pulse of 90 beats per minute, and a respiration rate of 22 cycles per minute. There was no cyanosis, clubbing, or icterus. The pain level was graded as 8/10 on the numerical pain rating scale (NPRS) on activity and 5/10 during rest.

Observational Results

The patient was evaluated while he was lying on his back with the right limb supported by a pillow and the hips and knees were extended. The patient was a 30-year-old man who has a mesomorphic built. Palpatory findings included the existence of Grade 3 tenderness and an increase in localized warmth. The table below mentions the range of motion (ROM) (Table 1). Movements at left knee and both ankles were pain-free, but right knee and hip movements were painful. Because of the fracture and pain, a straight-leg raise could not be performed. On neurological evaluation, all superficial and deep senses and reflexes were intact. A written informed consent was obtained from the patient.

**Table 1:** Assessment on Day 1 of physiotherapy treatment NA: not accessible

Joint	Movement	Right		Left	
		Active	Passive	Active	Passive
Hip	Flexion	NA	0°-30°	0°-110°	0°-120°
	Extension	NA	0°-15°	0°-15°	0°-20°
	Adduction	NA	0°-30°	0°-40°	0°-50°
	Abduction	NA	0°-15°	0°-25°	0°-30°
Knee	Flexion	NA	NA	0°-130°	0°-130°
	Extension	NA	NA	130°-0°	130°-0°
Ankle	Plantar Flexion	0°-25°	0°-50°	0°-45°	0°-50°
	Dorsi Flexion	0°-5°	0°-10°	0°-10°	0°-10°
	Inversion	0°-15°	0°-35°	0°-30°	0°-35°
	Eversion	0°-10°	0°-15°	0°-10°	0°-15°

Therapeutic interventions

Postoperative Management

The short-term goals were to reduce edema and pain, improve joint ROM, improve cardiopulmonary fitness, stimulate early mobility, and prevent pressure sores. The long-term goals were walking re-education, gait and balance training, maximizing patient functioning in performing activities of daily living (ADLs) as independently as possible. Tables 2, 3, and 4 show week-wise management of the patient.

**Table 2:** Summary of physiotherapy management from Week 1 to Week 4 Reps: repetitions, BD: twice a day, ROM: range of motion, AROM: active range of motion, SLR: straight leg raise, ADLs: activities of daily living

Sr. No	Goal	Intervention	Regimen
Week 1 (Day 1-2)			
1	Patient awareness	To educate the patient and his family about the importance of the physiotherapy	Counseling was done on effects of exercise, ambulation, and position
		Different positions for patient: initially semi-fowlers position, later, upright sitting at 90°	Positioning pillows was taught to patient and care givers
2	To prevent pulmonary complication & integumentary complications	Breathing exercises	10 reps x 1 set x BD
		Use of spirometer	10 reps x 1 set x BD
		Ankle pumps	10 reps x 1 set x BD
		Static exercises for hip and thigh muscles	10 reps x 1 set x BD
		AROM for unaffected leg	15 x 1 set x BD
3	Pain reduction	Application of cryotherapy at fracture site	7-8 mins each time x 4-5 times a day
Day 3-4			
1	To sustain thigh muscle power, to reduce joint rigidity, and to increase ROM	Active aided SLR 0°-5°	10 reps x 1 set x BD
2	To maintain hip abductor and adductor strength, to reduce joint rigidity, and improve ROM	Active aided hip abduction 0°-50 and adduction 0°-5°	10 reps x 1 set x BD
3	To avoid knee stiffness	Heel slides 0°-5°	10 reps x 1 set x BD
Day 5-7			
1	To maintain hip abductor and adductor strength	Active aided SLR 0°-10°; active aided hip abduction 0°-10° and adduction 10°-0°	10 reps x 1 set x BD
2	To reduce joint rigidity and increase mobility	Heel slides 0°-10°	10 reps x 1 set x BD
3	To initiate trunk and hip mobility	Single-leg bridging with support of unaffected leg	10 reps x 1 set x BD
Week 2			
1	To maintain thigh muscle power, to lessen joint tightness and increase ranges	Active SLR 0°-15°; heel slides 0°-15°	15 reps x 1 set x BD
2	To reduce joint rigidity and increase ranges and to sustain hip abductor and adductor strength	Active hip abduction 0°-10° and adduction 10°-0°	15 reps x 1 set x BD
3	Breathing exercises, ankle pumps, static exercises, single-leg bridging; active motions for unaffected extremity were continued.		15 reps x 1 set x BD
4	Pain reduction at fracture site	Cryotherapy	7-8 mins each time x 4-5 times a day
Weeks 3 and 4			
1	Advancement in active ROM	Active SLR 0-300; SLR in prone 0-100; abduction 0-100; heel slides 0- 300; active-assisted dynamic quadriceps 0-300	20 reps x 1 set x BD
2	To improve and maintain functional ROM, to enhance balance, reduce fall anxiety, build confidence and increase strength	Try bedside standing with assistance of a walker and also non-weight bearing walking with a walker	20 reps x 1 set x BD
3	Initiated weight bearing	Partial weight bearing exercise, pivot shifting	20 reps x 1 set x BD
4	Focusing on ADLs	Using western toilet, dressing and undressing himself	Started from Week 3
5	Breathing exercises, ankle pumps, static exercises, single-leg bridging; active movements for unaffected extremity were continued.		20 reps x 1 set x BD

**Table 3:** Summary of physiotherapy management from Week 5 to Week 8 AROM: active range of motion; SLR: straight leg raise; Reps: repetitions; TD: thrice a day; ADLs: activities of daily living

Sr. No	Goal	Intervention	Regimen
1	To improve AROM	SLR 0°-45°; SLR in prone 0°-30°; abduction 0°-15°; heel slides 0°-45°; dynamic quadriceps 0°-45°	20 reps x 1 set x TD
2	Improving endurance of hip and knee muscles	Self-resisted exercises for hip and knee muscles; isometric exercises for gluteus maximus and medius; resisted exercises of hip flexion, extension; abduction using weight cuffs and resistance band	20 reps x 1 set x TD
3	Mobilization of patient both in bed and out of bed	Patient was mobilized from long sitting to high sitting then to standing re-education then to walking re-education	With the aid of a walker, the patient commenced standing re-education on partial weight bearing. Started from Week 5.
4	To improve ADLs	Independent: toileting, personal hygiene	Started from Week 8

 

**Table 4:** Summary of physiotherapy management from Week 9 to Week 12 AROM: active range of motion, SLR: straight leg raise, Reps: repetition, TD: thrice a day; ADLs: activities of daily living

Sr. No	Goal	Intervention	Regimen
1	To improve AROM	SLR 0°-60°; SLR in prone 0°-20°; providing resistance; abduction 0°-30°; heel slides; 0°-130°; dynamic quadriceps 0°-70°	30 reps x 1 set x TD
		Exercises requiring resistance were performed with medium resistance using resistance band and dumbbells	30 reps x 1 set x TD
2	Weight bearing	Standing and walking re-education	Four-point gate using crutches was taught
3	Gait training	Marching on the same spot, hurdle clearance, stair climbing, backward walking, tandem walking, and clearing high steps was encouraged	Started from Week 9
4	To improve ADLs	Independence in all ADLs; avoiding limping gait	Care was taken by patient

Follow up and outcome

Post conclusion of physiotherapy intervention, the patient returned optimal functioning without complain of pain not decrease ROM. Patient reported improved ROM and muscle strength hence returned back to his previous work schedules. Tables 5 and 6 show the outcome measures.

**Table 5:** Manual muscle testing pre and post physiotherapy (grade out of 5) NA: not accessible

Muscle Group	Pre Physiotherapy		Post Physiotherapy	
	Right	Left	Right	Left
Hip Flexors	NA	NA	3	5
Hip Extensors	NA	NA	3	4
Hip Abductors	NA	NA	4	4
Hip Adductors	NA	NA	4	4
Knee Flexors	NA	NA	3	4
Knee Extensors	NA	NA	3	4
Ankle Plantar Flexors	3	4	4	5
Ankle Dorsiflexors	3	4	4	5

**Table 6:** ROM assessment after commencement of physiotherapy treatment ROM: range of motion; NA: not accessible

Joint	Movement	Right		Left	
		Active	Passive	Active	Passive
Hip	Flexion	NA	0°-30°	0°-110°	0°-120°
	Extension	NA	0°-15°	0°-15°	0°-20°
	Adduction	NA	0°-30°	0°-40°	0°-50°
	Abduction	NA	0°-15°	0°-25°	0°-30°
Knee	Flexion	NA	NA	0°-130°	0°-130°
	Extension	NA	NA	130°-0°	130°-0°
Ankle	Plantar Flexion	0°-25°	0°-50°	0°-45°	0°-50°
	Dorsi Flexion	0°-5°	0°-10°	0°-10°	0°-10°
	Inversion	0°-15°	0°-35°	0°-30°	0°-35°
	Eversion	0°-10°	0°-15°	0°-10°	0°-15°

## Discussion

In this case, the patient suffered from right hip pain, edema, limited ROM, and reduced strength. After assessing the patient, a treatment plan was created to address all of the symptoms. This plan included active and passive ROM exercises, muscular strengthening, and various modalities were used throughout the regime.

On the other hand, about 31% of the patients were using walking aids as a safety precaution, most of which consisted of canes. Additionally, many elderly people require some form of assistance [3]. Therefore, a conventional approach that emphasizes early weight bearing and ambulation in hip fractures is needed, which will compare how hip fractures are treated is reliable and effective. [9]. Following an iliac stress injury, using an antigravity treadmill for rehabilitation might assist in determining when it is safe to resume ground jogging [10]. In certain circumstances where it is preferable to have as minimal recovery time as possible, like in professional athletes and in individuals with extensively displaced fractures, surgical management may be necessary [11]. In my region, there are few studies looking at the critical role that rehabilitation intervention plays in fracture management. It is this gap that this study attempts to bridge. The patient was properly educated throughout the intervention with regard to what to expect, any probable difficulties, how to approach them, and the steps that would follow the surgery. To be better prepared to handle postoperative issues, the patient's psychological state should be improved. If the patient keeps a positive outlook, the chances of a timely return to normal activities and effective recovery enhances.

## Conclusions

Physiotherapy aids in enhancing daily living skills, physical function, and patients' independence. The patient made good improvements in part as a result of his desire to keep up his physical treatment and adequate care. This study concludes that combining definitive surgical techniques with early physiotherapy intervention hastens the clinical recovery of patients with fractures by reducing pain, restoring range of motion and muscle strength and aiding early ambulation/loading of the fractured limb. Following committed effort from both the physiotherapist and patient, the patient was fully rehabilitated as he could ambulate unaided.
